# Will Working Longer Enhance the Health of Older Adults? A Pooled Analysis of Repeated Cross-sectional Data in Japan

**DOI:** 10.2188/jea.JE20210030

**Published:** 2023-01-05

**Authors:** Takashi Oshio, Satoshi Shimizutani

**Affiliations:** 1 Institute of Economic Research, Hitotsubashi University, Tokyo, Japan; 2 JICA Ogata Sadako Research Institute for Peace and Development, Tokyo, Japan

**Keywords:** labor force participation, mental health, older adults, pensionable age, self-rated health

## Abstract

**Background:**

Encouraging older adults to continue working longer would be a realistic solution to the shrinking labor force, which is a result of the aging population. This study examined whether working longer improves the health of older adults.

**Methods:**

We used repeated cross-sectional data from 1,483,591 individuals aged 55–69 years collected from 11 waves of a nationwide population-based survey conducted in Japan from 1986 to 2016. We estimated pooled regression models to explain health outcomes by work status, controlling for potential endogeneity biases. Based on the estimation results, we conducted simulations to predict the health impact of policy measures that encourage older adults to participate in the labor force.

**Results:**

The regression analysis showed that work status had a mixed health impact. For example, work reduced the probability of poor self-rated health by 6.7 (95% confidence interval [CI], 6.2–7.2) percentage points and increased that of psychological distress by 12.2 (95% CI, 11.3–13.1) percentage points. The simulation results showed that raising both the mandatory retirement age and eligibility age for claiming public pension benefits to 70 years would increase the employment rate by 27.8 (standard deviation [SD], 4.2) percentage points among those aged 65–69 years, which would reduce their probability of poor self-rated health by 1.8 (SD, 0.4) percentage points and raise that of psychological distress by 4.1 (SD, 0.8) percentage points for that age group.

**Conclusion:**

The results suggest the need to pay attention to the health outcomes of policy measures that encourage older adults to work longer.

## INTRODUCTION

Under rapid population aging, a shrinking labor force presents an urgent challenge for many developed countries. One solution is to encourage older adults to work longer.^1^^,^^2^ Therefore, governments in advanced countries have implemented pension reforms, including increasing the eligibility age for claiming public pension benefits and other policy measures to enhance elderly labor force participation.^3^^,^^4^ However, a concern is whether older workers are healthy enough to continue working longer. This study examined the impact of work status on older adults’ health using data from a population-based survey conducted in Japan. Based on the estimation results, we conducted simulations to project the health impact of policy measures that enhance older adults’ labor force participation.

### Institutional background

**Figure 1.**
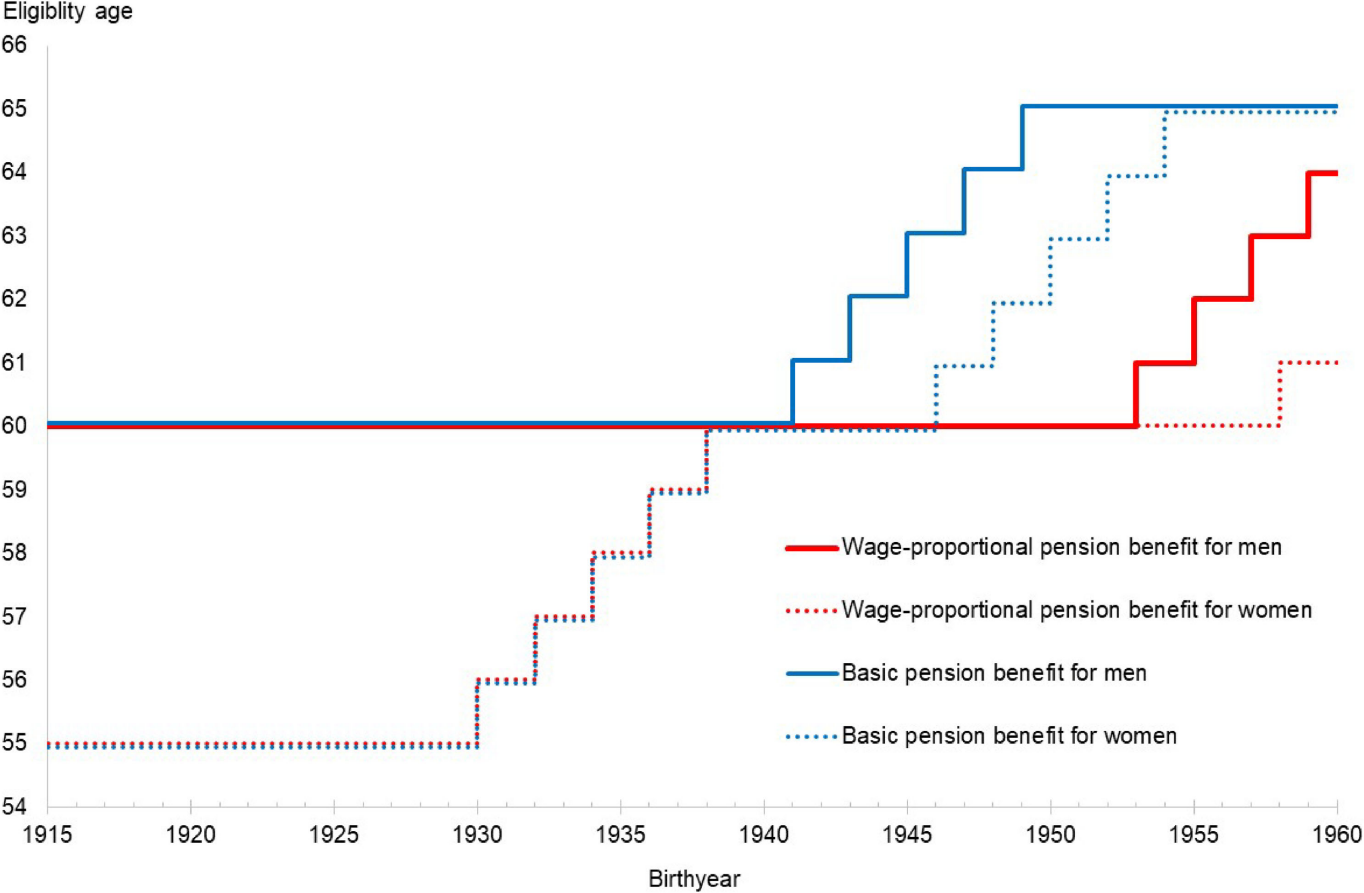
Eligibility ages for public pension benefits by sex and birth year

The public pension scheme has been a key determinant of retirement, and public pension benefits tend to disincentivize working.^5^^,^^6^ The eligibility ages of claiming pension benefits are exogenously determined according to respondents’ cohort, sex, and calendar years, allowing researchers to use them as exogenous variables to explain work status. The public pension benefit in Japan has a two-tier structure: the first tier is a flat-rate, basic benefit, and the second is a wage-proportional benefit.^7^ Self-employed workers receive only the basic benefit, whereas employed workers receive both. These two benefits have different eligibility ages, which the government has been gradually raising since 2001, as illustrated in Figure 1. In accordance with the extension of the pension eligibility ages, the government has been encouraging firms to raise the mandatory retirement age.^8^ The government recommended that firms formally define 60 years as the mandatory retirement age in 1986, and then required them to gradually raise it to 65 years in 2004. In 2020, the government recommended that firms keep hiring workers until 70 years of age. This mandatory retirement age is expected to be used as an exogenous variable for work status.

### Previous studies

An increasing number of studies have examined the impact of work on older adults’ health.^9^ Many studies indicated that employed older adults have better health outcomes in terms of self-rated health (SRH), multi-morbidity, physical functioning, activities of daily living (ADL), and others than unemployed/retired older adults.^10^^–^^13^ Furthermore, studies have provided evidence for the favorable impact on mental health of having a paid job.^14^^–^^17^ Equally important, studies have observed that health outcomes tend to improve after retirement,^18^^–^^20^ while other studies have expressed skepticism about the positive impact of retirement on health.^21^^,^^22^ One reason for these mixed results regarding the impact of work on older adults’ health may be the bidirectional relationship between work status and health.^23^^–^^25^ Healthier individuals are likely to work longer than unhealthier ones, rendering the causation from work to health ambiguous. If so, statistical analyses must explicitly address the endogeneity of work status.

### Research objectives

This study had two research objectives. First, we examined the association between work status and health among individuals aged 55–69 years, those most likely to be exposed to changes in mandatory retirement and/or pensionable ages. In the analysis, we controlled for potential biases arising from the endogeneity of work status. Second, based on the estimation results of these models, we conducted policy simulations to project how the extension of mandatory retirement and pensionable age to 70 years would increase the employment rate and affect health outcomes for those aged 55–69 years. Although there is no theoretical basis for a simulation study in observational epidemiology, the simulation results of this study can help quantitatively assess the impact of policy changes on older adults’ health.

## METHODS

### Study sample

The study was based on a dataset obtained from a nationwide population-based survey entitled “Comprehensive Survey of Living Conditions” (CSLC) conducted and released by the Ministry of Health, Labour and Welfare (MHLW). The CSLC has been conducted since 1986, and comprises a household survey conducted annually and a health and income/savings survey conducted once every 3 years. Samples of the CSLC were collected nationwide using a two-stage random sampling procedure. First, approximately 5,400 districts were randomly selected from around 940,000 national census districts. Second, approximately 290,000 households were randomly selected from each selected district according to its population size. The selected households were asked to provide information about each co-residing household member. Households were randomly selected in each wave; thus, the same household or individual may be repeatedly selected as a respondent only by chance.

We used repeated cross-sectional data collected from each of the 11 waves of the CSLC conducted every 3 years from 1986 to 2016, when data from both the household and health surveys were available. We restricted the study sample to individuals aged 55–69 years, or those impacted by the extension of the mandatory retirement and/or pensionable ages. After excluding respondents for whom essential information was missing, this study used the data of 1,483,591 individuals (706,192 men and 777,399 women). We obtained CSLC data with the MHLW’s permission. The CSLC was authorized by the Ministry of Internal Affairs and Communications (MIAC), which is in charge of all government surveys in Japan, from the statistical, legal, ethical, and other viewpoints in accordance with the Statistics Law in Japan. Hence, this study did not require ethics approval.

### Measures

#### Health outcomes

We considered five health outcomes obtained from responses in the CSLC: (1) SRH, (2) subjective symptoms, (3) ADL problems, (4) stress/anxiety, and (5) psychological distress. Regarding SRH, respondents were asked, “What is your current health status? Is it excellent, very good, good, fair, or poor?” We constructed binary variables for poor SRH, allocating 1 to *fair* or *poor*, and 0 otherwise. For subjective symptoms—ADL problems and stress/anxiety—the survey asked, respectively, “Have you been feeling ill owing to sickness or injury (that is, having any subjective symptom) for the past few days?” “Do you have any problem in daily life due to health?” and “Are you feeling stressed or anxious in daily life?” We constructed binary variables for each of having any subjective symptoms, ADL problems, and stress/anxiety, allocating 1 to those who answered yes to these questions, and 0 otherwise.

We also measured psychological distress using Kessler 6 (K6) scores.^26^^,^^27^ Earlier studies confirmed the reliability and validity of this score in psychological analyses of Japanese people.^28^^,^^29^ Respondents answer a six-item psychological distress questionnaire rated on a five-point scale (0 = *none of the time* to 4 = *all of the time*): “During the past 30 days, about how often did you feel a) nervous, b) hopeless, c) restless or fidgety, d) so depressed that nothing could cheer you up, e) that everything was an effort, and f) worthless?” We subsequently calculated the sum of the reported scores (range: 0–24) and defined it as the K6 score. K6 scores = 5+ indicate mood/anxiety disorder in the Japanese sample, as previously validated.^28^^,^^29^ We constructed a binary variable of psychological distress, allocating 1 to K6 scores = 5+ and 0 to others. SRH and subjective symptoms are reported over the entire sample period (1986–2016), whereas data on ADL problems, stress/anxiety, and psychological distress are available only from the 1989, 1995, and 2007 surveys, respectively.

#### Work status and other individual-level covariates

We constructed a binary variable for work status by allocating 1 to those who answered that they had a paid job in May (the survey was conducted in early June), regardless of job type. In addition to work status, we included as individual-level covariates binary variables for sex (female), marital status (married, unmarried, divorced/separated), each of six household types (living alone; husband and wife; husband and wife, and an unmarried child(ren); single parent and an unmarried child(ren); three-generation household; and other), and age and its squared value. We also included binary variables of each quartile of household expenditure and of having an owned house as proxies for household income level and assets, respectively.

#### Exogenous variables that predict work status

We considered six exogenous variables to predict work status, as discussed in the next section. The first was a binary variable for mandatory retirement age, allocating 1 if the age was 60 years or more in 1986–2003 or 65 years or more in 2007–2016. The second and third corresponded to eligibility for claiming basic and wage-proportional pension benefits, respectively. We constructed binary variables for each benefit, allocating 1 if the age was equal to or more than the eligibility age, which depended on sex and birth-year cohorts, as illustrated in Figure 1. We further considered three prefecture-level exogenous variables in each survey year: job offers/applicants ratio, share of workers in the primary industry (agriculture, farming, and fishing), and that in the secondary industry. The job offers/applicants ratio represents the prefecture’s overall labor market conditions, and the share of workers in the primary and secondary industries represents the prefecture’s economic structure. These prefecture-level variables were all downloadable from the MIAC website.^30^

### Statistical analysis

Following the descriptive analysis that compared the prevalence of each health outcome of workers and non-workers, we conducted a pooled regression analysis. Specifically, we estimated two probit models. The first was a conventional probit model to explain the probability of each health outcome by work, controlling for individual-level covariates and fixed effects at the prefecture and survey year levels. This probit model is expressed for individual *i* in prefecture *j* in survey year *t* as:

$$\begin{array}{rl} \text{Probit }({\text{Health}}_{jti}) & =\beta_{1}{\text{Work}}_{jti}+\sum_{j}\beta_{2j}{\text{Dpref}}_{j}\\ & \;+\sum_{t}\beta_{3t}{\text{Dyear}}_{t}+\sum_{k}\beta_{4k}{\text{Cov}}_{kjti}+\varepsilon_{jti} \end{array}$$

where *Health*, *Work*, *Dpref*, *Dyear*, *Cov*, and *ε* indicate health outcomes, work, prefecture indicators (46), survey year indicators (10), individual-level covariates, and an error term, respectively.

The second was a recursive bivariate probit model consisting of two probit equations that enable correlating the error terms with the binary dependent choice in one equation to an endogenous regressor in the other equation.^31^ In this study’s framework, the first equation intended to predict the choice of work status by a set of exogenous variables, and the second to predict the probability of each health outcome by work. The exogenous variables to predict work status in the first equation included (i) variables for mandatory retirement and pension eligibility, and (ii) prefecture-level job offers/applicants ratio and share of workers in primary and secondary industries. This recursive bivariate probit model is expressed for individual *i* in prefecture *j* in survey year *t* as:

$$\begin{array}{rl} \text{Probit }({\text{Work}}_{jti}) & =\sum_{m}\alpha_{1m}{\text{Zind}}_{mjti}+\sum_{n}\alpha_{2n}{\text{Zpref}}_{njt}\\ & \;+\sum_{j}\alpha_{3j}{\text{Dpref}}_{j}+\sum_{t}\alpha_{4t}{\text{Dyear}}_{t}\\ & \;+\sum_{k}\alpha_{5k}{\text{Cov}}_{kjti}+\varepsilon_{1jti}, \end{array}$$

$$\begin{array}{rl} \text{Probit }({\text{Health}}_{jti}) & =\beta_{1}{\text{Work}}_{jti}+\sum_{j}\beta_{2j}{\text{Dpref}}_{j}\\ & \;+\sum_{t}\beta_{3t}{\text{Dyear}}_{t}+\sum_{k}\beta_{4k}{\text{Cov}}_{kjti}+\varepsilon_{2jti}, \end{array}$$

where *Zind* and *Zpref* denote the individual- and prefecture-level exogenous variables, respectively, which are both specific to each survey year; *ε*_1_ and *ε*_2_ are error terms and assumed correlated. Note that *Zpref* is time-variant, unlike time-invariant *Dpref*, although both are prefecture-level variables. We included *Zpref* in the first equation and not in the second one, assuming that these variables, which are considered to reflect the labor market conditions and the industrial structure at the prefecture level, affect an individual’s work status but do not directly affect his/her health status.

Based on the estimation results of the recursive bivariate probit models, we then conducted a simulation to predict how the extension of mandatory retirement and pension eligibility ages to 70 years would affect the probabilities of work and health outcomes. We explain how to conduct the simulation in eMaterials 1.

## RESULTS

### Descriptive analysis

Table 1 summarizes the key features of the sample of this study. The employment rate decreased as age increased: from 73.1% in those aged 55–59 years to 38.4% in those aged 65–69 years. Compared to women, a higher proportion of men were working (72.6% vs 41.2%). Compared to non-workers, workers were younger (60.6 years vs 63.2 years), more likely married (84.6% vs 79.0%), more likely residing with an unmarried child/children (26.8% vs 19.6%), and had higher household expenditure (279.6 million JPY vs 258.0 million JPY).

**Table 1.** Key features of the study sample

	All (A)		Work (B)		Not work (C)	

		(% of total)		[B/A, %]		[C/A, %]
Men	706,192	(47.6)	512,356	[72.6]	193,836	[27.4]
Women	777,399	(52.4)	320,072	[41.2]	457,327	[58.8]
Aged 55–59 years	521,216	(35.1)	380,859	[73.1]	140,357	[26.9]
Aged 60–64 years	505,914	(34.1)	276,239	[54.6]	229,675	[45.4]
Aged 65–69 years	456,461	(30.8)	175,330	[38.4]	281,131	[61.6]

		(% of total)		(% of total)		(% of total)
Marital status						
Married	1,219,221	(82.2)	704,596	(84.6)	514,625	(79.0)
Unmarried	58,393	(3.9)	29,997	(3.6)	28,396	(4.4)
Divorced/separated	205,977	(13.9)	97,835	(11.8)	108,142	(16.6)
Household structure						
Alone	130,636	(8.8)	68,317	(8.2)	62,319	(9.6)
Husband and wife	475,421	(32.0)	237,681	(28.6)	237,740	(36.5)
Husband and wife and an unmarried child(ren)	351,380	(23.7)	223,448	(26.8)	127,932	(19.6)
Single parent and an unmarried child(ren)	55,487	(3.7)	29,801	(3.6)	25,686	(3.9)
Three-generation	303,455	(20.5)	173,615	(20.9)	129,840	(19.9)
Other	167,212	(11.3)	99,566	(12.0)	67,646	(10.4)
Having own house	1,286,980	(86.7)	723,600	(86.9)	563,381	(86.5)

	*Mean*	*SD*	*Mean*	*SD*	*Mean*	*SD*
Age	61.8	(4.3)	60.6	(4.1)	63.2	(4.1)
Household expenditure (monthly, thousand JPY)	270.2	(327.9)	279.6	(349.0)	258.0	(298.1)

Total	1,483,591		832,428		651,163	

SD, standard deviation.

Table 2 compares the prevalence of each health outcome by work status and age group, based on pooled cross-sectional data. The table shows that all health outcomes were better for workers than non-workers; for example, the prevalence of poor SRH was 13.0% among workers compared to 19.5% among non-workers in the entire sample. Furthermore, the rightmost columns indicate that workers were healthier than non-workers within each age group for all health outcomes.

**Table 2.** Prevalence (%) of health outcomes by work status and age group^a^

	All	Work	Not work	Difference	

		(A)	(B)	(A−B)	(SE)
Poor self-rated health (*N* = 1,352,788; study period, 1986–2016)					
All	15.8	13.0	19.5	6.5	(0.1)
Aged 55–59 years	14.4	12.7	19.1	6.4	(0.1)
Aged 60–64 years	15.6	12.8	19.0	6.1	(0.1)
Aged 65–69 years	17.8	13.9	20.2	6.3	(0.1)

Subjective symptom (*N* = 1,483,591; study period, 1986–2016)					
All	36.5	33.2	40.7	7.5	(0.1)
Aged 55–59 years	33.7	31.8	38.9	7.1	(0.1)
Aged 60–64 years	36.4	33.4	40.1	6.7	(0.1)
Aged 65–69 years	39.8	36.1	42.1	6.0	(0.1)

ADL problem (*N* = 1,218,037; study period, 1986–2016)					
All	14.7	11.8	18.5	6.7	(0.1)
Aged 55–59 years	12.4	10.7	17.4	6.6	(0.1)
Aged 60–64 years	14.6	11.9	17.9	6.0	(0.1)
Aged 65–69 years	17.3	13.9	19.4	5.5	(0.1)

Stress/anxiety (*N* = 967,703; study period, 1995–2016)					
All	44.9	44.6	45.4	0.8	(0.1)
Aged 55–59 years	48.7	48.1	50.4	2.3	(0.2)
Aged 60–64 years	44.2	43.2	45.5	2.3	(0.2)
Aged 65–69 years	41.7	39.5	43.1	3.6	(0.2)

Psychological distress (*N* = 446,515; study period, 2007–2016)					
All	26.4	25.2	28.2	3.0	(0.1)
Aged 55–59 years	29.4	28.3	33.7	5.4	(0.3)
Aged 60–64 years	25.6	23.8	28.5	4.6	(0.2)
Aged 65–69 years	24.1	21.5	25.9	4.5	(0.2)

ADL, activities of daily living; SE, standard error.

^a^*P* for trend for age groups were <0.001 for all cases.

### Regression analysis

Table 3 summarizes the estimation results of the probit and recursive bivariate probit models for poor SRH. The results are expressed as the marginal effect, which indicates an expected change in the dependent variable in response to a change in an explanatory variable from 0 to 1 if the dependent variable is a binary variable. The originally estimated coefficients (*β*) are provided in eTable 1.

**Table 3.** Estimation results of the probit and recursive bivariate probit models for predicting poor self-rated health^a^ (*N* = 1,352,788)

Regression model	Probit		Bivariate probit^b^			

Dependent variable	Poor self-rated health		Poor self-rated health		Work	

	*dy*/*dx*^c^	95% CI	*dy*/*dx*	95% CI	*dy*/*dx*	95% CI
Work	−0.064	(−0.066, −0.063)	−0.067	(−0.072, −0.062)		
Exogenous variables						
Mandatory retirement					−0.115	(−0.118, −0.112)
Eligibility of basic pension benefit					−0.092	(−0.095, −0.089)
Eligibility of wage-proportional pension benefit					−0.064	(−0.067, −0.061)
Prefecture-level variables						
Job offers ratio^d^					0.004	(−0.001, 0.009)
Primary industry^d^					0.279	(0.157, 0.402)
Second industry^d^					0.618	(0.496, 0.739)
Individual-level covariates						
Female	−0.016	(−0.017, −0.015)	−0.017	(−0.019, −0.015)	−0.297	(−0.299, −0.296)
Age^d^	−0.022	(−0.026, −0.017)	0.019	(0.016, 0.021)	−0.107	(−0.111, −0.104)
Age-squared/100^d^	0.019	(0.015, 0.023)	0.006	(0.003, 0.008)	−0.034	(−0.037, −0.031)
Marital status						
Unmarried	0.027	(0.024, 0.031)	0.027	(0.023, 0.031)	−0.166	(−0.171, −0.162)
Divorced/separated	0.005	(0.003, 0.008)	0.007	(0.004, 0.009)	−0.045	(−0.048, −0.042)
Household structure						
Alone	0.010	(0.007, 0.013)	0.009	(0.006, 0.013)	0.109	(0.105, 0.113)
Wife and husband and an unmarried child(ren)	−0.003	(−0.007, −0.013)	−0.004	(−0.006, −0.002)	0.053	(0.050, 0.055)
Single parent and an unmarried child(ren)	0.014	(0.010, 0.018)	0.012	(0.008, 0.017)	0.128	(0.123, 0.134)
Three-generation	0.002	(0.000, 0.004)	0.002	(0.000, 0.004)	0.077	(0.075, 0.080)
Other	0.026	(0.024, 0.028)	0.025	(0.023, 0.027)	0.076	(0.073, 0.078)
Household expenditure and housing						
Expenditure: 1st quartile (lowest)	0.014	(0.024, 0.028)	0.014	(0.012, 0.016)	0.004	(0.001, 0.006)
Expenditure: 2nd quartile	0.000	(0.024, 0.028)	0.000	(−0.001, 0.002)	−0.011	(−0.013, −0.009)
Expenditure: 3rd quartile	−0.001	(−0.003, 0.028)	−0.001	(−0.003, 0.001)	−0.018	(−0.020, −0.016)
Expenditure: unknown/unanswered	0.004	(0.024, 0.028)	0.004	(0.002, 0.007)	−0.027	(−0.030, −0.023)
Having own house	−0.039	(−0.041, −0.037)	−0.038	(−0.040, 0.036)	0.008	(0.005, 0.010)

CI, confidence interval.

^a^Controlled for exogenous variables, individual-level covariates, and prefecture- and survey year-level fixed effects.

^b^The Wald test for the null hypothesis of no correlation between the errors of the two probit models for health and work was rejected (*P* < 0.001).

^c^Marginal effect. For a binary explanatory variable, this indicates an expected change in the probability of poor self-rated health in response to a change from 0 to 1 of the explanatory variable.

^d^Continuous variables (others were binary variables).

The conventional probit model shows that work reduced the probability of poor SRH by 6.4 percentage points. In comparison, the recursive bivariate probit model indicated that the negative impact on the probability of poor SRH was 6.7 percentage points, slightly higher than that observed in the probit model. The probability of work was reduced by 11.5, 9.2, and 6.4 percentage points, respectively, if the age was equal to or more than the mandatory retirement age, eligibility age for the basic pension benefit, and eligibility age for the wage-proportional benefit, respectively. We also observed how prefectural-level exogenous variables affected the probability of work. The job offers/applicants ratio was not related to the probability of work, whereas the shares of the primary and secondary industries were positively associated with it; 1 percentage-point increase in primary and secondary industry shares rose the probability of work by around 0.3 and 0.6 percentage point, respectively.

We repeated a similar regression analysis for other health outcomes. Table 4 summarizes the estimated impact of work on them in terms of the marginal effect. The originally estimated coefficients (*β*) are provided in eTable 2. The recursive bivariate probit models showed that work reduced the probability of subjective symptoms and ADL problems by 14.2 and 9.7 percentage points, respectively, for the all sample, and the estimated impacts were larger than those from the conventional probit models (4.9 and 6.6 percentage points), as in the case of poor SRH. The recursive bivariate probit models also indicated that work increased the probability of stress/anxiety and psychological distress. Notably, the direction of the impact on stress/anxiety and psychological distress was reversed from negative (−1.7 and −4.4 percentage points) in the conventional probit model to positive (16.4 and 12.2 percentage points) in the recursive bivariate model. The table also shows a similar pattern of estimated effects of work status on each health outcome for men and women. The results of recursive bivariate models show that the favorable impacts of work on SRH and ADL were somewhat larger among women (−9.2 percentage points among women compared to −5.6 percentage points among men for SRH, and −12.6 percentage points among women compared to −8.7 for ADL).

**Table 4.** Estimated associations of work with selected health outcomes^a^

Health outcome	Probit		Bivariate probit^b^		*N*

	*dy*/*dx*^c^	95% CI	*dy*/*dx*	95% CI	
All					
Poor self-rated health	−0.064	(−0.066, −0.063)	−0.067	(−0.072, −0.062)	1,483,591
Subjective symptoms	−0.049	(−0.050, −0.047)	−0.142	(−0.149, −0.136)	1,483,591
ADL problems	−0.066	(−0.067, −0.064)	−0.097	(−0.102, −0.091)	1,218,037
Stress/anxiety	−0.017	(−0.019, −0.014)	0.164	(0.157, 0.171)	967,703
Psychological stress	−0.044	(−0.047, −0.041)	0.122	(0.113, 0.131)	446,515

Men					
Poor self-rated health	−0.083	(−0.085, −0.081)	−0.056	(−0.062, −0.049)	645,618
Subjective symptoms	−0.064	(−0.067, −0.062)	−0.143	(−0.151, −0.135)	706,192
ADL problems	−0.089	(−0.091, −0.087)	−0.087	(−0.093, −0.081)	584,614
Stress/anxiety	−0.035	(−0.039, −0.032)	0.165	(0.156, 0.174)	468,350
Psychological stress	−0.060	(−0.064, −0.056)	0.115	(0.104, 0.126)	219,236

Women					
Poor self-rated health	−0.048	(−0.050, −0.046)	−0.092	(−0.100, −0.083)	707,170
Subjective symptoms	−0.036	(−0.038, −0.034)	−0.149	(−0.159, −0.139)	777,399
ADL problems	−0.046	(−0.048, −0.044)	−0.126	(−0.135, −0.118)	633,423
Stress/anxiety	−0.005	(−0.008, −0.002)	0.159	(0.148, 0.170)	499,353
Psychological stress	−0.030	(−0.034, −0.026)	0.118	(0.104, 0.132)	227,279

ADL, activities of daily living; CI, confidence interval.

^a^Controlled for exogenous variables, individual-level covariates, and prefecture- and survey year-level fixed effects.

^b^The Wald test for the null hypothesis of no correlation between the errors of the two probit models for health and work was rejected for all cases (*P* < 0.001).

^c^An expected change in the probability of each health outcome in response to a change from 0 to 1 of the explanatory variable.

### Simulation analysis

Based on the estimated results of the recursive bivariate probit models, we simulated how the extension of mandatory retirement and pension eligibility ages to 70 years would affect the probabilities of work and health outcomes. Table 5 compares the simulation results across the sample (aged 55–69 years) and three age groups (55–59, 60–64, and 65–69 years). In 2016, the recommended mandatory retirement also increased to 65 years.

**Table 5.** Simulated impacts on employment and health outcomes of raising mandatory retirement and public pension eligibility ages to 70 years

		Aged 55–69 years		Aged 55–59 years		Aged 60–64 years		Aged 65–69 years	

		*Mean*	*SD*	*Mean*	*SD*	*Mean*	*SD*	*Mean*	*SD*
Work	Actual	61.4	(48.7)	78.9	(40.8)	65.3	(47.6)	44.9	(49.7)
	Predicted (A)	61.3	(21.4)	75.5	(14.4)	68.2	(17.1)	45.3	(18.4)
	Simulated (B)	74.7	(14.3)	76.1	(13.6)	75.3	(13.8)	73.1	(15.0)
	Impact (B−A)	13.3	(12.7)	0.7	(2.1)	7.1	(5.0)	27.8	(4.2)

Poor self-rated health	Actual	14.6	(35.3)	14.3	(35.0)	14.5	(35.2)	15.0	(35.7)
	Predicted (A)	14.6	(4.0)	13.4	(3.6)	14.3	(3.9)	15.9	(4.1)
	Simulated (B)	13.8	(3.8)	13.4	(3.6)	13.8	(3.8)	14.1	(3.8)
	Impact (B−A)	−0.9	(0.8)	0.0	(0.1)	−0.4	(0.3)	−1.8	(0.4)

Subjective symptoms	Actual	30.7	(46.1)	29.0	(45.4)	30.1	(45.9)	32.3	(46.8)
	Predicted (A)	31.0	(7.3)	28.3	(6.2)	30.3	(7.1)	33.7	(7.5)
	Simulated (B)	29.2	(6.7)	28.2	(6.1)	29.4	(6.9)	29.9	(7.0)
	Impact (B−A)	−1.8	(1.7)	−0.1	(0.3)	−1.0	(0.7)	−3.8	(0.7)

ADL problems	Actual	15.0	(35.7)	14.0	(34.7)	15.0	(35.7)	15.8	(36.5)
	Predicted (A)	15.2	(5.7)	13.3	(4.8)	14.8	(5.5)	17.0	(5.8)
	Simulated (B)	13.9	(5.1)	13.3	(4.8)	14.0	(5.3)	14.2	(5.2)
	Impact (B−A)	−1.4	(1.3)	−0.1	(0.2)	−0.8	(0.5)	−2.8	(0.8)

Stress/anxiety	Actual	45.0	(49.7)	49.6	(50.0)	45.4	(49.8)	41.1	(49.2)
	Predicted (A)	45.2	(9.0)	48.5	(7.9)	45.6	(8.7)	42.3	(9.2)
	Simulated (B)	47.7	(8.9)	48.6	(8.0)	47.4	(8.9)	47.1	(9.5)
	Impact (B−A)	2.5	(2.1)	0.2	(0.6)	1.8	(1.0)	4.8	(0.8)

Psychological stress	Actual	26.5	(44.2)	30.0	(45.8)	26.4	(44.1)	23.8	(42.6)
	Predicted (A)	27.3	(7.1)	29.8	(6.5)	27.7	(6.9)	25.0	(7.1)
	Simulated (B)	29.4	(7.2)	30.0	(6.5)	29.3	(7.2)	29.1	(7.7)
	Impact (B−A)	2.1	(1.9)	0.2	(0.6)	1.6	(0.9)	4.1	(0.8)

ADL, activities of daily living; SD, standard deviation.

As seen here, the extension of the mandatory retirement and pension eligibility age to 70 years increased the probability of work, especially among those aged 65–69 years (27.8 percentage points), who would have received pension benefits under the current public pension scheme and are most likely to have left the workplace after the current mandatory retirement. Compared with this age group, the impact on work status was smaller for younger age groups.

**Figure 2.**
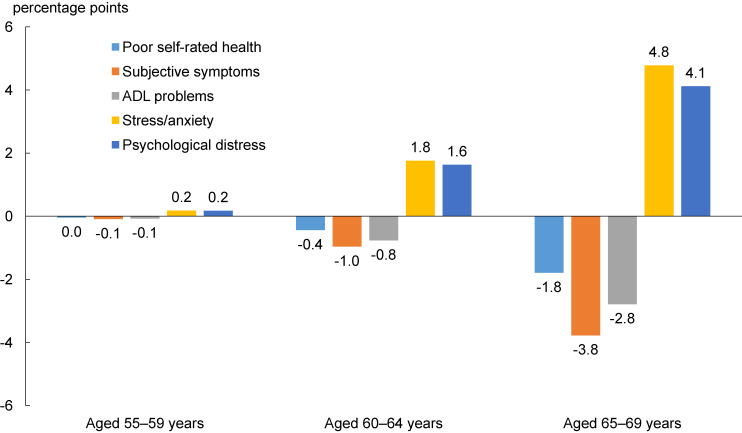
Simulated impacts on health outcomes of raising the eligibility ages for mandatory retirement and public pension to 70 years ADL, activities of daily living.

Correspondingly, each health outcome was affected by enhanced employment. The impact concentrated on those aged 65–69 years. The probability of poor SRH was reduced by 1.8 percentage points for that age group, compared with a more limited or absent reduction for the younger groups. The probabilities of subjective symptoms and ADL problems also declined, especially among those aged 65–69 years. In contrast, the probabilities of stress/anxiety and psychological distress increased in response to enhanced employment, especially among those aged 65–69 years (4.8 and 4.1 percentage points, respectively). Figure 2 illustrates the simulated impacts on health outcomes of raising mandatory retirement and public pension eligibility ages to 70 years among the three age groups.

## DISCUSSION

We examined the impact of working longer on the health of older adults based on large-scale, repeated cross-sectional data obtained from a population-based social survey conducted in Japan. The results of the regression models were mixed. After controlling for endogeneity biases, we found that working tended to reduce the risk of reporting poor SRH, subjective symptoms, and ADL problems in line with previous studies that positively associated work and health in old age.^10^^–^^13^ A comparison of the results between the conventional and recursive bivariate models suggests that endogeneity biases tend to underestimate the favorable impact of work on health. Meanwhile, the impact on mental health was negative, judging from the results of stress/anxiety and psychological distress. This finding contrasts those of previous studies that showed that work is good for mental health.^14^^–^^17^ These observations and those for general health outcomes suggest that the endogeneity issue may be more serious for mental than physical health. The results highlight the importance of their bidirectional associations, especially for mental health.^24^^,^^25^ Furthermore, the observed negative impact of work on mental health may be aligned with previous observations on the positive effect of retirement on mental health.^18^^,^^19^^,^^22^

The simulation results showed that extending the mandatory retirement and pensionable age to 70 years would raise the employment rate by nearly 30 percentage points among individuals aged 65–69 years. The results also show that the increase in older adults’ labor force participation will be accompanied by mixed changes in their health, that is, reduced probabilities of poor SRH, subjective symptoms, and ADL problems, and enhanced risks of stress/anxiety and psychological distress. These results imply that policymakers should be cautious about the health outcomes, especially in terms of mental health, of policy measures that encourage older adults to work longer. Although enhanced labor force participation may promote more flexible working styles and improve physical health, older adults forced to work longer will be exposed to more psychological pressure. The results also suggest that extending the mandatory retirement or pensionable age will have a more limited impact on the health of younger age groups, because their current working status is less sensitive to these policy changes.

The health impact of policy measures to encourage older adults to work longer may depend on institutional settings, especially whether individuals are forced or motivated to keep working after reaching the mandatory retirement or pensionable age. For example, if older adults are allowed to keep working without a substantial reduction in pension benefits, or even with some tax incentives, then the negative impact on mental health may be more limited than what the results of this study suggest. Moreover, it is likely that the beneficial impact on health may dominate if individuals can flexibly adjust their working style with a combination of wage and pension benefits.

This study has several limitations. First, the cross-sectional analysis could not fully dismantle the bidirectional relationship between work status and health,^24^^,^^25^ and information about previous health was not available from the dataset. Second, this study did not distinguish between types of work, especially full-time and part-time work, and ignored the possibility of their change over time. The impact on health may likely differ between employed and self-employed workers^32^^,^^33^ and also be confounded by the type of occupation, which is likely to affect physical and mental health as well as retirement age. Third, the analysis should be expanded to cover social activities other than paid jobs, such as volunteer work, which has a substantial impact on the health of older adults.^12^^,^^16^^,^^17^ Fourth, family relationships including marital and parental status and caregiving for older parents, can confound the relationship between work status and health.^23^^,^^34^ Fifth, this study focused on the overall health impact on older adults’ health in the Japanese institutional context, requiring caution in generalizing the observed results. Last, our regression models did not account for the hierarchical data structure consisting of three levels (1,483,591 individuals at level 1, nested within 11 survey years at level 2, nested within 47 prefectures at level 3), although we controlled for prefecture- and year-level fixed effects.

Despite these limitations, we can conclude that staying longer in the labor force is generally good for physical health but adversely impacts mental health. In addition, the simulation results suggest that attention be paid to the ambiguous health outcomes of policy measures that promote older adults’ extended labor force participation.

## SUPPLEMENTARY MATERIAL

eMaterials 1. Simulation procedure

eTable 1. Original estimation results of the probit and recursive bivariate probit models for predicting poor self-rated health^a^ (*N* = 1,352,788)

eTable 2. Estimated associations of work with selected health outcomes^a^
