# Supplementary material for: Will Working Longer Enhance the Health of Older Adults? A Pooled Analysis of Repeated Cross-sectional Data in Japan
Source: J Epidemiol. 2023 Jan 5;33(1):15–22. doi: 10.2188/jea.JE20210030 (PMC9727210; doi:10.2188/jea.JE20210030)
Supplement: Supplementary file 1 [file je-33-015-s001.pdf]

eMaterial 1. Simulation procedure

We conducted a simulation to estimate the impact of raising both the mandatory retirement age and eligibility age for claiming public pension benefits to 70 years on work and health as follows.

Recursive bivariate probit model

1. We estimated the recursive bivariate probit model:

$$\text{Probit}\ (Work_{jti}) = \sum_m \alpha_{1m} Zind_{mjti} + \sum_n \alpha_{2n} Zpref_{njt} + \sum_j \alpha_{3j} Dpref_j + \sum_t \alpha_{4t} Dyear_t + \sum_k \alpha_{5k} Cov_{kjti} + \varepsilon_{1jti}, \quad (1)$$
$$\text{Probit}\ (Health_{jti}) = \beta_1 Work_{jti} + \sum_j \beta_{2j} Dpref_j + \sum_t \beta_{3t} Dyear_t + \sum_k \beta_{4k} Cov_{kjti} + \varepsilon_{2jti}, \quad (2)$$

to jointly predict the probabilities of work and each (unfavorable) health outcome.

Probability of work

2. We predicted the probability of work before the policy changes, $P_0\ (Work)$, using the actual values of each explanatory variable and estimated coefficients in the equation predicting the probability of work and applying a cumulative standard normal distribution (Φ):

$$P_0\ (Work_{jti}) = \Phi[\sum_m \hat{\alpha}_{1m} Zind_{mjti} + \sum_n \hat{\alpha}_{2n} Zpref_{njt} + \sum_j \hat{\alpha}_{3j} Dpref_j + \sum_t \hat{\alpha}_{4t} Dyear_t + \sum_k \hat{\alpha}_{5k} Cov_{kjti}], \quad (3)$$

where $\hat{\alpha}$s were the estimated coefficients corresponding to αs in Eq. (1).

3. We simulated the probability of work after the policy changes, $P_1\ (Work)$, by replacing all the binary variables of (i) mandatory retirement, (ii) eligibility for basic pension benefit, and (iii) eligibility for wage-proportional pension benefit with *zero*, with other explanatory variables unchanged. Thus, by replacing all *Zind*s in Eq. (3) with zero, we calculated

$$P_1\ (Work_{jti}) = \Phi[\sum_n \hat{\alpha}_{2n} Zpref_{njt} + \sum_j \hat{\alpha}_{3j} Dpref_j + \sum_t \hat{\alpha}_{4t} Dyear_t + \sum_k \hat{\alpha}_{5k} Cov_{kjti}], \quad (4)$$

for each individual. If $\hat{\alpha}_1$s are negative, we can expect $P_1\ (Work_{jti}) > P_0\ (Work_{jti})$ by comparing Eqs. (3) and (4). For example, if the probability of work is negatively related to mandatory retirement, abolishing it is expected to raise the probability of work.

4. We computed the difference between the means of P_1 (*Work*) and P_0 (*Work*) for each age group in 2016 to assess the policy impact on the employment rate for each age group.

Probability of health outcome

5. For each health outcome, we predicted its probability before the policy changes, P_0 (*Health*), using the estimated coefficients and actual values of each explanatory variable on the right-hand side of Eq. (2), which predicted the probability of the health outcome. Here, for the variable of work, we used the predicted benchmark probability of work, P_0 (*Work*), rather than the actual value of the binary variable of work. As with the probability of work, we predicted the probability of a health outcome by applying the cumulative standard normal distribution. Thus, we calculated

$$P_0\,(Health_{jti}) = \Phi[\hat{\beta}_1 P_0(Work_{jti}) + \textstyle\sum_j \hat{\beta}_{2j} Dpref_j + \sum_t \hat{\beta}_{3t} Dyear_t + \sum_k \hat{\beta}_{4k} Cov_{kjti}], \quad (5)$$

where $\hat{\beta}$s were the estimated coefficients corresponding to βs in Eq. (2).

6. We simulated the probability of work after the policy changes, P_1 (*Health*), by replacing P_0 (*Work*) with P_1 (*Work*), with other explanatory variables unchanged. Thus, we calculated

$$P_1\,(Health_{jti}) = \Phi[\hat{\beta}_1 P_1(Work_{jti}) + \textstyle\sum_j \hat{\beta}_{2j} Dpref_j + \sum_t \hat{\beta}_{3t} Dyear_t + \sum_k \hat{\beta}_{4k} Cov_{kjti}]. \quad (6)$$

If $\hat{\beta}_1$ is negative and $P_1\,(Work_{jti}) > P_0\,(Work_{jti})$, then we can expect $P_1\,(Health_{jti}) < P_0\,(Health_{jti})$ by comparing Eqs. (5) and (6). For example, if the probability of health outcome is negatively related to work, an enhanced probability of work (caused by the policy change) is expected to reduce the probability of health outcome.

7. We compared the difference between the means of P_0 (*Health*) and P_1 (*Health*) for each age group in 2016 to assess the impact of policy changes on the probability of health outcomes.

eTable 1. Original estimation results of the probit and recursive bivariate probit models for predicting poor self-rated health[a] (N = 1,352,788)

Regression model	Probit		Bivariate probit[b]			
Dependent variable	Poor self-rated health		Poor self-rated health		Work	
	β	95% CI[c]	*β*	95% CI	*β*	95% CI
Work	-0.270	(-0.276, 0.265)	-0.281	(-0.303, 0.259)		
Exogenous variables						
Mandatory retirement					-0.350	(-0.358, -0.341)
Eligibility of basic pension benefit					-0.279	(-0.288, -0.270)
Eligibility of wage-proportional pension benefit					-0.194	(-0.203, -0.185)
Prefecture-level variables						
Job offers ratio					0.012	(-0.003, 0.026)
Primary industry					0.850	(0.478, 1.222)
Second industry					1.879	(1.509, 2.249)
Individual-level covariates						
Female	-0.068	(-0.073, -0.062)	-0.071	(-0.080, -0.063)	-0.904	(-0.909, -0.899)
Age	-0.092	(-0.111, -0.073)	0.078	(0.066, 0.090)	-0.327	(-0.337, -0.317)
Age-squared	0.079	(0.064, 0.095)	0.024	(0.013, 0.034)	-0.104	(-0.113, -0.094)
Marital status						
Unmarried	0.115	(0.024, 0.131)	0.113	(0.097, 0.129)	-0.506	(-0.520, -0.492)
Divorced/separated	0.022	(0.003, 0.033)	0.028	(0.017, 0.039)	-0.137	(-0.146, -0.127)
Household structure						
Alone	0.043	(0.007, 0.056)	0.040	(0.026, 0.054)	0.332	(0.319, 0.344)
Wife and husband and an unmarried child (ren)	-0.012	(-0.007, -0.005)	-0.017	(-0.025, -0.010)	0.160	(0.153, 0.166)
Single parent and an unmarried child (ren)	0.059	(0.010, 0.076)	0.052	(0.034, 0.070)	0.391	(0.375, 0.406)
Three-generation	0.010	(0.000, 0.018)	0.010	(0.002, 0.018)	0.235	(0.228, 0.242)

Other	0.110	(0.024,	0.120)	0.106	(0.096,	0.116)	0.230	(0.222,	0.239)
Household spending and housing									
Expenditure: 1st quartile	0.058	(0.024,	0.066)	0.060	(0.052,	0.068)	0.011	(0.004,	0.018)
Expenditure: 2nd quartile	0.001	(-0.024,	0.009)	0.002	(-0.006,	0.010)	-0.033	(-0.040,	-0.026)
Expenditure: 3rd quartile	-0.005	(-0.013,	0.003)	-0.004	(-0.012,	0.004)	-0.054	(-0.061,	-0.047)
Expenditure: unknown/unanswered	0.018	(0.024,	0.030)	0.019	(0.007,	0.031)	-0.082	(-0.092,	-0.071)
Owned house	-0.163	(-0.171,	-0.155)	-0.160	(-0.168,	-0.152)	0.023	(0.016,	0.031)

CI, confidence interval.

[a]Controlled for exogenous variables, individual-level covariates, and prefecture- and survey year-level fixed effects.

[b]The Wald test for the null hypothesis of no correlation between the errors of the two probit models for health and work was rejected ($P < 0.001$).

eTable 2. Estimated associations of work with selected health outcomes[a]

Health outcome	Probit		Bivariate probit[b]		*N*
	β	95% CI[c]	*β*	95% CI	
All					
Poor self-rated health	-0.270	(-0.276, -0.265)	-0.281	(-0.303, -0.259)	1,483,591
Subjective symptoms	-0.132	(-0.136, -0.127)	-0.388	(-0.406, -0.370)	1,483,591
ADL[d] problems	-0.291	(-0.297, -0.285)	-0.428	(-0.450, -0.405)	1,218,037
Stress/anxiety	-0.043	(-0.049, -0.037)	0.429	(0.409, 0.448)	967,703
Psychological distress	-0.135	(-0.144, -0.126)	0.378	(0.350, 0.406)	446,515
Men					
Poor self-rated health	-0.356	(-0.365, -0.347)	-0.238	(-0.265, -0.211)	645,618
Subjective symptoms	-0.177	(-0.184, -0.170)	-0.397	(-0.420, -0.374)	706,192
ADL problems	-0.395	(-0.405, -0.386)	-0.387	(-0.415, -0.359)	584,614
Stress/anxiety	-0.092	(-0.100, -0.083)	0.433	(0.408, 0.457)	468,350
Psychological distress	-0.194	(-0.208, -0.180)	0.367	(0.331, 0.402)	219,236
Women					
Poor self-rated health	-0.197	(-0.205, -0.190)	-0.378	(-0.413, -0.342)	707,170
Subjective symptoms	-0.095	(-0.101, -0.089)	-0.401	(-0.428, -0.373)	777,399
ADL problems	-0.205	(-0.213, -0.197)	-0.550	(-0.585, -0.515)	633,423
Stress/anxiety	-0.013	(-0.020, -0.005)	0.415	(0.384, 0.445)	499,353
Psychological distress	-0.090	(-0.102, -0.078)	0.354	(0.311, 0.397)	227,279

ADL, activities of daily living; CI, confidence interval.

[a]Controlled for exogenous variables, individual-level covariates, and prefecture- and survey year-level fixed effects.

[b]The Wald test for the null hypothesis of no correlation between the errors of the two probit models for health and work was rejected ($P < 0.001$).
